# Effects of a Cereal and Soy Dietary Formula on Rehabilitation of Undernourished Children at Ouagadougou, in Burkina Faso

**DOI:** 10.1155/2012/764504

**Published:** 2011-11-28

**Authors:** Zoenabo Douamba, Marina Martinetto, Virginio Pietra, Salavatore Pignatelli, Fabian Schumacher, Jean-Baptiste Nikiema, Jacques Simpore

**Affiliations:** ^1^Centre Médical Saint Camille de Ouagadougou, Ouagadougou, Burkina Faso; ^2^Centre de Recherche Biomoléculaire Pietro Annigoni, Saint Camille-CERBA/LABIOGENE-Ouagadougou, Université de Ouagadougou, 03 BP 7021 Ouagadougou 03, Burkina Faso; ^3^University of Brescia, Piazza Spedali Civili 1, 25123 Brescia, Italy

## Abstract

The New Misola consists of millet soybean, peanut, vitamins, minerals, and industrial amylase. Our objective is to demonstrate that porridge made from local grains and legumes restores the nutritional balance of malnourished children. The study was carried on 304 malnourished children aged 6–48 months including 172 girls and 132 boys from Saint Camille Medical Centre. At the beginning, these malnourished children had a WHZ *z*-score of −3.10 and a WAZ *z*-score of −3.85, which reflected, according to WHO, a severe malnutrition. After eight weeks of nutritional rehabilitation, a normal WHZ of −1.41 was obtained. These children recovered more than those in a similar study performed in 2006 with the old formula of Misola. This study shows that malnutrition remains a public health problem in Burkina Faso. It should be necessary that public health services and the epidemiologists work in synergy with nutritionists and “*nutrigenetics*” in order to combat malnutrition efficiently.

## 1. Introduction

Malnutrition is a major public health problem. Indeed, undernutrition affects several millions of people worldwide, mainly children under five years. FAO estimates that a total of 925 million people are undernourished in 2010 compared with 1.023 billion in 2009; that is higher than before the food and economic crises of 2008-2009 and higher than the level that existed when world leaders agreed to reduce the number of hungry by half at the World Food Summit in 1996 [1]. Thus, in developing countries, nearly one in five is undernourished, while, in Africa, it is one in three. In West Africa, many studies report that, in hospitals, children with severe malnutrition have a mortality rate of 20% compared to 4% for those who are not [2, 3].

Malnutrition is a condition resulting from deficiency or excess of one or more essential nutrients. Undernutrition that occurs most often in developing countries leads in children under 5 years to stunting or delayed growth, emaciation, and underweight. It mainly causes diseases such as marasmus and kwashiorkor [4]. Overnourishment causes obesity, diabetes, and high cholesterol, and undernutrition is a risk factor for cardiovascular disease [5].

Like other Sahelian countries, Burkina Faso is confronted each year with significant risks for food security and nutritional deficiencies. Malnutrition represents 1/3 of the direct and indirect causes of child mortality at pediatric age. In Burkina Faso, prevalence of acute diseases linked to nutritional deficiencies was 21.2% in 2003 [6], while in 2009 chronic malnutrition rate in children less than 5 years was 35.1%, which constitutes more than 330,000 children suffering from acute malnutrition and over 1,000,000 children with chronic malnutrition [7]. 

For many children, lack of access to enough food is not the only cause of malnutrition. The lack of nutritional quality, poor feeding practices, and parasitic, bacterial, and viral infections act synergistically in the worsening and aggravation of the nutritional problems [8, 9]. However, primitive man lived basically on picking and hunting and fed mainly on vegetables, fruits, and grains he met on his way. Despite this food insecurity, he found solutions to nutritional problems in nature. Today, with the introduction of cash crops at the expense of food crops, overexploitation of soils due to overpopulation, industrialization, urbanization, and the development of slums and suburbs adjacent to large cities, children from poor families live in a nutritional imbalance. 

The management of cases of severe malnutrition in developing countries through the use of porridges composed of flour of local grain and legumes in order to restore nutritional balance for the infant is today a priority target for developing countries. In this purpose, for over 15 years, in the Center for Recovery and Nutritional Education (CREN) of the Saint Camille Medical Centre (CMSC), each year, almost 800 malnourished children with marasmus (M), kwashiorkor (K), or both (M, K) have been nutritionally recovered thanks to a porridge prepared from local grains and legumes: *Pennisetum glaucum* (millet), *Soja hispida* (soja), *Arachis hypogaea* (peanut). This porridge is prepared from a flour called “Misola” whose production, in Burkina Faso, is supported by a nonprofit organization: the Burkinabe Association of Misola Units.

 Phytochemical and biochemical studies of Misola flour show that it contains different types of proteins, glucoses, and carbohydrate elements that promote nutrition and human health [4]. From 2010, a change has occurred in the composition of the flour Misola. The New Misola (NM) is enriched with vitamins, minerals, and industrial amylase. In addition, in this new flour, the amount of sugar has risen from 2.5 kg to 3.5 kg for 25 kg. 

The main objective of this study was to demonstrate that the porridge made from local grains and legumes can help to restore nutritional balance. It is in this purpose that the New Misola was used in the CMSC to save malnourished children suffering from marasmus or kwashiorkor, or both. 

The specific objectives of this research were to (i) search for causes of malnutrition in the city of Ouagadougou, (ii) estimate, from the anthropometric parameters of malnourished children, the degrees of severity through *z*-scores calculations, (iii) use the New Misola to recover nutritionally malnourished children, and, finally, (iv) compare the results of this study that used the New Misola (NM) with those of the study carried out in 2006 on the old formula of Misola.

## 2. Subjects and Methods

### 2.1. Study Patients

The study was conducted in the CREN (Center for Recovery and Nutritional Education) of Saint Camille Medical Centre (CMSC), where about 800 malnourished children are followed every year. The study recruited all the 310 malnourished children attending this CREN from November 2010 to April 2011. Among them, 304 children, aged 6 to 48 months (average age 13.84 ± 6.68 months) including 172 girls and 132 boys, have followed the protocol of nutritional rehabilitation for 8 weeks. For 6/310 children, we stopped the research protocol because their health status required a reference to the national pediatric hospital. Among the 304 children no death was observed during the eight weeks of nutritional rehabilitation. 

At the beginning of this study, undernourished children were anorexic and many of them had diarrhea and were treated with nose-gastric (NG) rehydration according to the CMSC protocol [10]. They were taken off NG feeding before being selected for this study, since this condition had sufficiently improved to allow moving on to oral feeding. Criteria of exclusion were refusal to participate in the study, while criteria of discontinuation of participation were abandonment, death, and the interruption of treatment at the centre during the study. All studied children were undernourished according to the *z*-score criteria, recommended by WHO and UNICEF: (i) *z*-score inferior or equal to −3 standard difference corresponds to severe malnutrition; (ii) *z*-score between −3 standard difference and −2 standard difference corresponds to moderate malnutrition; (iii) *z*-score greater than −2 standard difference corresponds to a normal nutritional status. The ages of undernourished children were confirmed by their birth notebooks.

### 2.2. Anthropometric Parameters

Weight, height, brachial perimeters (BPs), and head perimeters (HPs) of the children were recorded. The weight of the children was recorded once a week from the day of admission to the CREN with a 10-gram sensitivity balance. The brachial perimeter (BP) is the mid-upper-arm circumference (MUAC). It is measured at the midpoint between the tips of the shoulder and elbow using an MUAC tape. The MUAC of children aged 6–59 months shows the degree of malnutrition: BP < 11.0 cm indicates severe acute malnutrition; 11.0 cm < BP < 12.5 cm corresponds to a moderate malnutrition; 12.5 cm < BP < 13.5 cm corresponds to a risk of malnutrition; 13.5 < BP corresponds to a satisfactory nutritional status [11]. 

The measure of head circumference (head perimeter); in children, head growth has been used by healthcare providers as a marker of brain well-being, because an abnormal rate of growth could suggest a pathological disorder requiring diagnosis and possible treatment, for example, hydrocephalus, psychosocial problems, and craniosynostosis. Measure Head Circumference as the name implies, occipital frontal circumference (OFC) is a measurement of the circumference of the head around the occiput, or posterior aspect, of the skull, to the most anterior portion of the frontal bone. An accurate head circumference measure is obtained with a flexible nonstretchable measuring tape [12]. The height of children <2 years was measured by resting the child in the supine position; in those children >2 years, height was measured in the upright position (as described by Simpore et al. [13]). The nutritional status, evaluated by brachial perimeters, was compared to Jelliffe's classification [14], considering that it varies little for children <4 years. HAZ (height for age *z*-score), WHZ (weight for height *z*-score), and WAZ (weight for age *z*-score) parameters were calculated according to the references of the National Center for Health Statistics (NCHS) [15]. At the CMSC, numbers from 1 to 4 drawn from the norms of Stuart and Stevenson [16] are used as measures to show the stages of severity of malnutrition. The number 1 is the primitive stage (M1 or K1), number 2 shows a moderate stage (M2 or K2), number 3 shows the severe stage (M3 or K3), and number 4 shows a very severe stage (M4 or K4).

### 2.3. Evaluation of Results

The evaluation of the nutritional status of the children has been made according to the nutritional indices (as described by Simpore et al., [4]). The weight for age index expressed in *z*-score (WAZ) or weight insufficiency indicates a global malnutrition affecting both the linear growth and the weight increment. The height for age index expressed in *z*-score (HAZ) or growth delay is an index indicating chronic malnutrition provoked by an extended reduction of food consumption and by repeated pathologic episodes. Emaciation or weight loss expressed by the weight for height index (WHZ) indicates a slightly less malnutrition status or weight deficit due to a decrease or slowdown of regular growth.

### 2.4. Preparation and Administration of the New Misola (NM)

The flour is produced by the handicraft production unit (Unité de Production Artisanale: UPA) of the CMSC, which has been operating since 1998 and is part of the network of UPAs acknowledged by the Misola Association (http://www.misola.org), which is responsible for the quality control of the product every year. 

The mothers of the undernourished children fed on New Misola (NM) were given weekly rations of 1500 grams, three bags, each containing 500 grams of flour. The daily quantity in grams of MN porridge to provide to children with malnutrition is obtained by the following formula: (*P* + 250)/4, where “*P*” is the weight in decagrams. This formula is done by the Misola Association. We specify that the Misola is a dietary supplement it is not a substitute for breastfeeding which is highly recommended for malnourished young children. The NM flour is a mixture of millet, soya, peanut kernel, sugar, salt, vitamins and industrial amylase. In 100 g of NM used, we have 405 kcal of energy, 16.8 g of proteins, 10.1 g of lipids, and 63 g of carbohydrates, minerals, and vitamins. The preparation of the NM was carried out according to traditional customs, namely, 60 grams of flour and 200 mL of water were mixed and boiled over a low fire, mixing for 5-6 minutes. Each mother has to cook it, 4 times a day (6:30, 10:30, 14:30, and 18:30), in order to feed her child. After this preliminary phase they continued to administer the mixture at home. Each day they accompanied their children to the CREN to monitor weight and other anthropometric parameters and deliver the 24-hours diet recall sheet to CREN (as described by Simpore et al., [4]).

### 2.5. Survey

The parents of the malnourished children have freely agreed to answer a questionnaire relating to their jobs, their educational level, and number of children living and deceased.

### 2.6. Statistical Analysis

Demographic and clinical profile were memorized in Excel sheet and analyzed by standard software SPSS-17 and EpiInfo-6. Statistical significance was set at *P* < 0.05. The Chi^2^ test was used to compare the proportions of different parameters of the study, whereas the *t*-test was used to compare the nutritional status of children in the 2006 survey with those of 2011.

## 3. Results

This study was carried out on 304 undernourished children utilizing New Misola.  Table 1 shows the information on the occupation and the level of school training of the parents of these undernourished children. We note that 284 (93.4%) mothers were illiterate and only 12 (3.95%) fathers were integrated in the public service. In this study, 264 (86.8%) mothers were housewives. 

Table 2 presents the different kinds of malnutrition: 92.43% of these children had marasmus, a severe malnutrition (M3), and 2.30% had moderate grade marasmus (M2) at the beginning of the investigation. The number of malnourished children at M3 decreases significantly with age: the age group number 1 (36.65%); number 2 (32.03%); number 3 (14.23%).

Table 3 shows the biochemical composition for 100 grams of used Misola at the CMSC. The lipid composition is represented by palmitic, linoleic, oleic, *γ*-linolenic, stearic and palmitoleic acids [4].

Males' age, weight, height, head perimeter (HP), and brachial perimeter (BP) were greater than females with respective significance: *P* = 0.115 (NS), *P* = 0.002, *P* < 0.001, *P* < 0.001, and *P* = 0.061 (NS) (see Table 4).

Table 5 compares the anthropometric parameters (age, weight, height, and brachial perimeter (BP)) of this research with those of Simpore et al. [4]. In all parameters, we have differences that are statistically significant: age (*P* = 0.004), weight (*P* = 0.013), height (*P* = 0.004) and BP (*P* < 0.001). 

Table 6 shows the anthropometrics parameters of the children at the beginning and at the end of our study and those of Simpore et al., [4]. In this research, the *z*-scores identified at the beginning of the study and those of the end have statistic difference significance: WHZ1 versus WHZ2 (*P* < 0.001) and WAZ1 versus WAZ2 (*P* < 0.001). It was also found statistic difference significance when comparing *z*-scores from this study and those of Simpore et al., [4]: WHZ1 versus WHZ1 (*P* < 0.001); WAZ2 versus WAZ2 (*P* = 0.033). We do not consider the parameters size and age (HAZ) because the study lasted two months and the sizes of the children have not increased significantly. 

Table 7 reveals that, before the nutrition rehabilitation, (55.26%) 168 of malnourished children had WHZ < −3 and therefore a severe condition. After 8 weeks of nutritional rehabilitation, there was only (06.58%) 20 children with WHZ < −3 and (63.81%) 194 children have progressed with a normal WHZ > −2. Similarly, for WAZ, we had (85.53%) 250 children who had a WAZ < −3, and, after nutritional rehabilitation, there was only (38.16%) 116 children with WAZ < −3. 

## 4. Discussion 

The New Misola (NM) we have used for the nutritional rehabilitation of the children was composed of 60% of small millet, 20% of soya beans, and 10% of groundnuts which contain proteins, glucose, and lipids. Moreover, it had been enriched with vitamins, carbohydrates, minerals, and industrial amylase. 

Treatment compliance was excellent, and none of the children dropped out. The mothers reported that the children accepted the meal of NM. They attended weekly appointments, but only the first and the last visit (8 weeks) were considered in the final evaluation. 

After eight weeks of study, children treated with NM, appeared clinically improved; their weight increased. This study enabled us to show that the malnutrition of pediatric aged children is directly related to the level of their parents' level of education and their occupation. In fact, most of their mothers were illiterate (93.42%) and housewives (86.84%) while only 14.14% of their fathers were literate and 3.95% had a monthly salary. Moreover, their parents had several other children (41.4% of mothers had more than 3 living children), and some of them were even deceased. Emphasize that 20.4% of parents had at least one child died and 7.2% had more than 3 children died. These elements could reveal family penury and poverty which would partly account for such malnutrition of the living children when they are less than 5 years old (Table 1). But we have no statistical data indicating that these dead children have died from malnutrition. In addition we know that other studies have shown that, among malnourished children, some had viral infections (rotavirus, adenovirus, HIV) [17], bacterial and parasitic infections that cause diarrhea and dehydration, leading them to death. 

We notice that the majority of these children were in stage M3 which corresponds to a severe marasmus (Table 2). At the beginning of the study the malnourished children had a *z*-score of weight and height WHZ = −3.10 and a *z*-score of weight and age WAZ = −3.85 which meant, according to the WHO criteria, a severe malnutrition (Table 6). According to the *z*-score WHZ, the children of this study were much more malnourished than those of Simpore et al. in 2006 [4] (−3.10 ± 0.94 versus −1.73 ± 2.51) with *P* < 0.001. 

However, eight weeks after their nutrition with New Misola, we score a normal WHZ of −1.41 which is similar to the results obtained by Simpore et al. [4] (*P* = 0.118). Other studies carried out on malnourished children with other foods, such as that [18] of Ekpo et al. Abidoye and Nwachie, [19], and Kwena et al., [20]. With this study, in 8 weeks, the children have recovered 1700 grams or 30.36 grams/day, whereas, in the study by Simpore et al. [4], children recuperated 20 grams/day. Now, when we consider the WAZ, our children had the same level of emaciation as for the study carried out by Simpore et al., [4] (*P* = 0.067). Nevertheless, the children in this study had recovered more than those of Simpore et al. [4] (*P* = 0.033). That could be caused by the addition of vitamins, minerals, and industrial amylase in the Misola flour. 

The flour of the New Misola is basically composed of millet, soya beans, and groundnuts as detailed previously. Apart from the proteins, glucose, and lipids, millet is a grain that contains microelement such as magnesium, calcium, iron zinc, copper, and manganese. Groundnuts contain glucose, protides, lipids, sodium, potassium, manganese, calcium, iron, zinc, and so forth, while soya beans contains calcium, iron, magnesium, phosphorus, potassium, sodium, vitamins (A, B1, B2, B3, B5, B6, B12, C, E), folic acid, linoleic acid, alpha-linolenic acid, isoflavone, and so forth. 

The inflationary trend, “the cost of living” in Burkina Faso, makes it almost impossible for many poor families to afford animal protein from meat, fish, and egg for their children. This has led us to the search for an alternative (vegetable protein) to animal protein in human diets. The use of plant protein can serve as a complement to animal protein in human diets so as to increase the total protein intake [21]. Among the plant protein sources, soya beans is identified as one of the best because of its relatively high protein content and amino acid profile that is only low in sulphur-containing amino acids [22]. Several studies have shown that soybean can be grown in almost all parts of West Africa [23–25]. The regular consumption of the Misola porridge which contains soya beans helps to complement, in the undernourished children, the low intake of animal protein. 

Therefore, we understand the reason why the New Misola porridge can nutritionally rehabilitate undernourished children. The Misola, through its elements of composition (zinc, iron, and selenium), could reestablish also in the malnourished children the depressed immune system [26, 27].

## 5. Conclusion

This study shows that malnutrition remains a public health problem in Burkina Faso and over the world. The consequences of malnutrition represent a global problem, which affects morbidity as well as mortality. Awaiting the enrolment of these undernourished children in rehabilitation protocols, those in charge of public health services and epidemiologists should work in synergy with nutritionists and nutria-genetics in order to combat malnutrition efficiently [4]. According to the instructions that the mothers received, involvement of the families of the undernourished children and of the whole community is essential to control the great prevalence of malnutrition in African countries. 

Indeed, whole grains and legumes bring to the human being energy and the needed micronutrients for his or her metabolic homeostasis. Without the external contribution of these fundamental elements produced by these vegetables, the human being would be unable to synthesize them for his or her own metabolic processes. At the present time of globalization, the world seems to have become a big village. The kitchens of the international community must review their diet by basing them upon grains and legumes that are sources of a sound nutrition. Moreover, it would be very important to promote the science of nutrition and to develop the nutria-genetics science in order to cast new foundations of nutrition for the future, taking into account the requirements of population's genetics.

##  Ethical Committee

The Ethics Committee of the Saint Camille Medical Centre approved this protocol of study and authorized each person, after oral consent, to accept this study.

## Figures and Tables

**Table 1:** Parents' jobs, educational level.

	Occupation					Level of training	
	Traders	Handicraft	Wage-earner	Housewife	Pupil	Illiterate	Literate
Father	272	20	12			261	43
	89.47%	6.58%	3.95%			86.86%	14.14%
Mother	24	14		264	2	284	20
	7.89%	4.61%		86.84%	0.66%	93.42%	6.58%

**Table 2:** Different kinds of malnutrition with the 304 children according to the age groups.

Age group	Months	M1	M2	M3	K1	K2, M3	Total
1	6 to 9	2/4	2/7	103/281	2/3		109/304
		50.00%	28.57%	36.65%	66.67%		35.85%
2	10 to 14			90/281	1/3	2/9	93/304
				32.03%	33.33%	22.22%	30.59%
3	15 to 19		5/7	40/281			45/304
			71.43%	14.23%			14.80%
4	20 to 48	2/4		48/281		7/9	57/304
		50.00%		17.09%		77.78%	18.75%

	Total	4	7	281	3	9	304

*χ* ^2^ : 1 → 2; *P* = 0.248,  *χ* ^2^ : 1 → 3; *P* < 0.001,  *χ* ^2^ : 1 → 4; *P* < 0.001,  *χ* ^2^ : 2 → 3; *P* < 0.001,*χ* ^2^ : 2 → 4; *P* < 0.001,  *χ* ^2^: 3 → 4; *P* = 0.353.

M1: marasmus phase 1; M2: marasmus phase 2; M3: marasmus phase 3; K1 and K2: Kwashiorkor phases 1 and 2.

**Table 3:** Biochemical composition of Old Misola (OM) and new Misola (NM) produced at Saint Camille Medical Centre.

Biochemical composition	Old Misola	New Misola
Protein (g)	15	16.8
Lipid (g)	11	10.1
Glucides (g)	61	63
Added sugar	10% of the formula	13% of the formula
vitamins	—	0.8% of the formula
minerals	3 g	0.8% of the formula
Amylase	Germinated sorghum flour	800 mg Industrial Amylase^1^
Calories/kcal/100 g	425	405

^1^Amylases are enzymes that hydrolyze starch, that is, fragment chains cooked starches to provide soluble sugars. We switch from a thick consistency to a liquid without loss of nutrients. Amylase in the liquefaction has nothing in common with the dilution with water. The slurry becomes sweeter.

Amylases can be obtained locally from germinated cereals: malt sorghum, millet, or corn.

Indeed, we used to germinate the seeds and produce amylase, but this product was quickly spoilt by heat. That is why we are using industrial amylase now, as it is stable.

Dried, ground, and sieved, the malt is added in very small quantities in the hot porridge after cooking (as it is partly destroyed by heat).

The malt can also be added to the flour before cooking, but it is then necessary to add ten to fifteen times more. Amylase industry is not destroyed by cooking and is very powerful. Thus, it can be incorporated into the flour, in minute quantities before cooking.

**Table 4:** Median anthropometric parameters of the 304 children according to sex at beginning and at the end of the study.

	Females (172)			Males (132)			All children (304)			
Parameters	Initial mean	End mean	*P*: I → E	Initial mean	End mean	*P*: I → E	Initial mean	End mean	*P*: I → E	*P*: Females_i → Males_i
Age (months)	13.31 ± 6.64	15.73 ± 6.53		14.53 ± 6.72	16.82 ± 6.53		13.84 ± 6.68	16.20 ± 6.53		
Weight (kg)	5.64 ± 1.29	7.28 ± 1.43	<0.001	6.10 ± 1.27	7.88 ± 1.45	<0.001	5.84 ± 1.30	7.54 ± 1.47	<0.001	0.002
Height (cm)	68.78 ± 6.16	70.2 ± 6.21	0.134	70.85 ± 6.26	72.71 ± 6.05	0.085	69.68 ± 6.27	71.29 ± 6.25	0.026	0.004
HP (cm)	42.02 ± 2.06	43.14 ± 2.16	0.001	43.23 ± 2.10	44.30 ± 1.86	0.002	42.55 ± 2.16	43.65 ± 2.11	<0.001	<0.001
BP (cm)	10.46 ± 1.10	12.66 ± 0.91	0.001	10.71 ± 1.21	12.7 ± 0.93	0.001	10.57 ± 1.15	12.68 ± 0.91	<0.001	0.061

BP: brachial perimeter; HP: head perimeter.

**Table 5:** Anthropometric parameters according to the two studies.

		2011 study			2006 study		*t*-test: ^∧^→ *: *P *
Parameters	Females (172) Mean	Males (132) Initial mean	All children (304)^∧^	Females (286) Mean	Males (264) Initial mean	All children (550)*	
Age (months)	13.31 ± 6.64	14.53 ± 6.72	13.84 ± 6.68	15.64 ± 8.08	15.01 ± 6.87	15.30 ± 7.41	0.004
Weight (kg)	5.64 ± 1.29	6.10 ± 1.27	5.84 ± 1.30	5.82 ± 1.17	6.28 ± 1.36	6.07 ± 1.29	0.013
Height (cm)	68.78 ± 6.16	70.85 ± 6.26	69.68 ± 6.27	68.07 ± 6.13	68.43 ± 7.48	68.27 ± 7.13	0.004
BP (cm)	10.46 ± 1.10	10.71 ± 1.21	10.57 ± 1.15	10.75 ± 1.13	10.99 ± 1.25	10.88 ± 1.20	<0.001

BP: Brachial perimeter.

**Table 6:** Nutritional status at the beginning 1 and end of the study 2.

	304 children with New Misola 200 g/day, 2011	170 children with Misola 200 g/day [4]	*P*
WHZ1 1 → 2	−3.10 ± 0.94	−1.73 ± 2.51	<0.001
	*P* < 0.001	*P* = 0.035	
WHZ2	−1.41 ± 1.08	−1.14 ± 2.64	0.118
WAZ1 1 → 2	−3.85 ± 0.87	−4.01 ± 0.98	
	*P* < 0.001	*P* < 0.001	0.067
WAZ2	−2.75 ± 0.89	−2.95 ± 1.12	0.033

WHZ1, WAZ1: *z*-scores of the beginning; WHZ2, WAZ2: *z*-scores of the end.

WHZ1: weight for height *z*-score at beginning of the study; WHZ2: weight for height *z*-score at the end of the study; WAZ1: weight for age *z*-score at the beginning of the study; WAZ2: weight for age *z*-score at the end of the study.

**Table 7:** Percentage of nutritional rehabilitation of children according to *z*-scores.

Stage	WHZ			WAZ		
	WHZ1	WHZ2	*P*	WAZ1	WAZ2	*P*
*z*-score > −2	32 (10,53%)	226 (74,34%)	<0.001	0 (0%)	64 (21,05%)	—
−3 < z-score < −2	104 (34,21%)	58 (19,08%)	0.035	44 (14,47%)	124 (40,79%)	<0.001
*z*-score < −3	168 (55,26%)	20 (06,58%)	<0.001	260 (85,53%)	116 (38,16%)	<0.001

WHZ1: weight for height *z*-score at beginning of the study; WHZ2: weight for height *z*-score at the end of the study; WAZ1: weight for age *z*-score at the beginning of study; WAZ2: weight for age *z*-score at the end of the study.
